# A divergent Tbx6-related gene and Tbx6 are both required for neural crest and intermediate mesoderm development in *Xenopus*

**DOI:** 10.1016/j.ydbio.2010.01.013

**Published:** 2010-04-01

**Authors:** Elizabeth M. Callery, Gerald H. Thomsen, James C. Smith

**Affiliations:** a The Wellcome Trust/Cancer Research UK Gurdon Institute and Department of Zoology, The Henry Wellcome Building of Cancer and Developmental Biology, University of Cambridge, Tennis Court Road, Cambridge CB2 1QN, UK; b Department of Biochemistry and Cell Biology, Stony Brook University, Stony Brook, NY 11794-5215, USA; c MRC National Institute for Medical Research, The Ridgeway, Mill Hill, London, NW7 1AA, UK

**Keywords:** Tbx6r, Tbx6, T-box, Neural crest, *Xenopus*, Development, Evolution

## Abstract

T-box family transcription factors play many roles in Metazoan development. Here we characterise Tbx6r, a unique Tbx6 paralogue isolated from the amphibian *Xenopus*. The evolution and developmental integration of this divergent T-box gene within the vertebrates reveals an unexpected level of plasticity within this conserved family of developmental regulators. We show that despite their co-expression, *Tbx6* and *Tbx6r* have dissimilar transcriptional responses to ligand treatment, and their ability to activate ligand expression is also very different. The two paralogues have distinct inductive properties: Tbx6 induces mesoderm whereas Tbx6r induces anterior neural markers. We use hybrid proteins in an effort to understand this difference, and implicate the C-terminal regions of the proteins in their inductive specificities. Through loss-of-function analyses using antisense morpholino oligonucleotides we show that both Tbx6 paralogues perform essential functions in the development of the paraxial and intermediate mesoderm and the neural crest in *Xenopus*. We demonstrate that Tbx6 and Tbx6r both induce *FGF8* expression as well as that of pre-placodal markers, and that Tbx6 can also induce neural crest markers via a ligand-dependent mechanism involving FGF8 and Wnt8. Our data thus identify an important new function for this key developmental regulator.

## Introduction

The T-box encodes a highly conserved DNA-binding domain of approximately 180 amino acids. It was first identified through work on the *Brachyury* (*T*) mouse mutant and subsequently discovered in many proteins throughout the Metazoa (Adell et al., 2003; Agulnik et al., 1995; Kispert and Herrmann, 1993; Pflugfelder et al., 1992). The T-box gene family has undergone significant diversification during evolution: thirteen, seventeen and twenty members have been identified in cnidarians, mammals and nematodes respectively (Andachi, 2004; Naiche et al., 2005; Yamada et al., 2007). T-box genes perform numerous developmental functions, which in many cases have been evolutionarily conserved, as illustrated by the requirements for *Tbx5* in heart and limb development, *Tbx4* in limb formation, *Tbx1* in pharyngeal development, and both *Brachyury* and *Tbx6* in posterior mesoderm patterning in many vertebrates (Bongers et al., 2004; Martin and Kimelman, 2008; Naiche et al., 2005; Piotrowski et al., 2003). The developmental importance of T-box genes in vertebrates is underscored by the severity of mutant phenotypes in mice: *Brachyury* mutants are homozygous lethal (Chesley, 1935) and there is a dramatic conversion of mesodermal to neural tissue in *Tbx6* mutants, resulting in embryos with three neural tubes (Chapman and Papaioannou, 1998).

Much research on the T-box family has emphasized the functional conservation of orthologous members throughout evolution, so the origin and characterisation of a novel T-box protein would be of particular interest. In this paper, we characterise *Tbx6r*, a unique *Xenopus* T-box gene, which has no apparent orthologues in the genomes of any other vertebrate class. A partial clone of this gene was independently isolated as Xtbx6r (Yabe et al., 2006); however, we show that this cDNA encodes a truncated protein, lacking the N-terminus.

We have analysed Tbx6r from an evolutionary developmental context, examining how this gene evolved, its likely ancestor, its biological activities, and its developmental functions. We show that *Tbx6r* evolved through genomic locus duplication rather than through retrotransposition and that this locus is transcriptionally active in both *X. laevis* and *X. tropicalis*. We find that although *Tbx6r* expression is similar to that of *Tbx6*, its inductive properties are very different and we use hybrid proteins to identify the regions that define this functional specificity.

Using antisense morpholino oligonucleotides (MOs) to deplete Tbx6r, we demonstrate that this protein is required for development of the somitic musculature, intermediate mesoderm and neural crest. These discoveries prompted us to analyse the possible involvement of its syn-expression homologue, *Tbx6*, in these processes, revealing novel functions of this important developmental regulator in the patterning of the neural crest and intermediate mesoderm. Significantly, we find that Tbx6 can induce neural crest in a non-autonomous manner requiring both FGF8 and Wnt8 signalling.

## Materials and methods

### Cloning of full-length *Tbx6r* cDNA

A cDNA clone encoding Tbx6r was identified by a TBLASTN search of the *Xenopus laevis* EST database using *Xbra* as a query (Genbank accession number AW641903). To identify the full-length sequence, 5′ RACE was performed using the BD SMART RACE cDNA amplification kit (Clontech). The Genbank Accession number for the full-length sequence is EF015907. The complete ORF was subcloned into the expression vector pCS2+.

### Comparative sequence analysis

A multiple sequence alignment of T-domain sequences was performed with Tcoffee and a phylogenetic tree was constructed with the PhyML maximum likelihood program (Guindon and Gascuel, 2003) using the WAG substitution model with 100 bootstrap datasets (http://atgc.lirmm.fr/phyml/).

### Genomic cloning of the Tbx6r locus

Genomic DNA from *Xenopus laevis* tadpole tails was isolated using the DNeasy kit (Qiagen) and amplified using RedTaq (Sigma). Amplification conditions were: denaturation at 95 °C for 1 min; 30 amplification cycles of 95 °C for 1 min, 52 °C for 1 min, 68 °C for 1 min and 30 s; elongation at 70 °C for 10 min.

### Southern blotting

Genomic DNA was isolated using Qiagen Genomic Tips. After digestion with either EcoRI or XbaI, 10 μg genomic DNA was loaded per lane on a 0.8% agarose/1X TAE gel and subsequently blotted onto Hybond XL using alkaline transfer, according to the manufacturer's instructions. A PCR-generated DNA fragment corresponding to nucleotides 712-1362 of the Tbx6r cDNA was used as a template in the generation of a random-primed probe. Pre-hybridization and the overnight hybridization steps were performed at 62 °C in Rapid-Hyb Buffer (GE Healthcare). The blot was washed at 58 °C prior to autoradiography, with two 15 min washes each of 2X SSC, 0.5X SSC and 0.2X SSC, each wash containing 0.1% SDS.

### Western blotting

Embryos were homogenized in 1% NP40, 150 mM NaCl, 20 mM Tris pH 7.5, 2 mM EDTA, 50 mM NaF, 1 mM sodium pyrophosphate plus proteinase inhibitors (Roche) and Freon-extracted prior to SDS-PAGE. HRP-labelled antibodies were detected using SuperSignal West Pico Chemiluminescent Substrate (Pierce).

### Molecular biology techniques

Part of the 5′ UTR and the complete ORF of Tbx6r and Tbx6 were cloned into pCS2-MT (Turner and Weintraub, 1994), to generate Tbx6r–MT and Tbx6–MT, respectively. Site-directed mutagenesis of Tbx6r–MT was used to create the Tbx6r–M1R and M20R constructs. The Tbx6r–Tbx6 and Tbx6–Tbx6r hybrid clones were created in pCS2+ by ligating an N-terminal fragment, consisting of the N-terminus and T-box, together with a C-terminal fragment of the respective proteins, as depicted in Fig. 2A. A three amino acid AGL linker was introduced between the two fragments during construction of the hybrids. Tbx6-∆C was created by introducing a stop codon at the end of the T-domain, at K284, by site-directed mutagenesis.

Capped mRNA for embryo microinjection was synthesised using mMessage mMachine (Ambion). Synthesis of digoxigenin-labelled RNA probes, in situ hybridisation and immunohistochemistry were performed as described (Sive et al., 2000).

RNA was extracted from embryonic samples using Trizol Reagent (Invitrogen) and treated with DNAse I (Roche) before reverse transcription with AMV-RT (Roche). cDNA was phenol:chloroform extracted prior to real-time quantitative PCR on a LightCycler (Roche).

Sequences of antisense morpholino oligonucleotides (‘morpholinos’) targeted against *X. laevis* Tbx6r were: (MO1); 5′-CTGAGTCCAGACAGGGACAGGCAGT-3′ (MO2); 5′-GTGAACATGCCCACCCATCTCTCTC-3′ (MO3); 5′-CTTGTCACTCACCTTCCACTCTTAG-3′ (MO4). The *X. tropicalis* morpholinos were 5′-ACCAGGCAATTGGCACCTACCTGCC-3′ (Tbx6r) and 5′-GGGAATTCAGATCTGCCAAAG-3′ (Tbx6). The *X. laevis* Tbx6 splicing morpholino (T6sp1) was 5′-CACCTGATCGTCTCACCTGCCAGAC-3′ and that directed against the translational start site (Tbx6 ATG) was 5′-AGCTCAGAGTGGTACATGGCTGC-3′. The control morpholino was the GeneTools standard. The FGF8 and Wnt8 morpholinos have been characterised previously (Fletcher et al., 2006; Li et al., 2006).

### Embryological techniques

Embryos were fertilized *in vitro* using standard methods (Sive et al., 2000) and staged as described (Nieuwkoop and Faber, 1975). Animal caps were dissected at stage 9 and cultured in agarose-lined dishes in 0.7X MMR/0.1% BSA containing 50 μg/ml gentamicin until collection. Ten animal caps were collected for each experimental sample.

## Results

### Tbx6r is a divergent Tbx6 paralogue that arose through gene duplication

We identified *Tbx6r* by BLAST searching the *Xenopus laevis* EST database for previously uncharacterised sequences containing a T-domain. We performed 5′ RACE to identify the full-length sequence, which encodes a 449 amino acid protein. A partial clone, lacking the first 19 amino acids but otherwise identical, was isolated independently (Yabe et al., 2006) and named *Xtbx6r* (*Xtbx6*-*r*elated) because its T-domain shares 53% identity with Tbx6. The proteins show no homology outside the T-domain (Fig. 1C, Yabe et al., 2006) . Phylogenetic analysis indicates that Tbx6r is a highly divergent paralogue of the Tbx6 sub-family, which includes Tbx6, Tbx16 and MGA in *Xenopus* (Fig. 1A). The long branch-length of *Tbx6r* on the phylogenetic tree means we cannot ascertain, based solely on sequence comparison, from which of these loci *Tbx6r* arose.

To investigate the evolutionary origins of Tbx6r in more detail, we examined its genomic structure. This analysis was facilitated by the existence of highly conserved splice sites within the DNA-binding domains of T-box family members (Campbell et al., 1998; Wattler et al., 1998). Amplification of genomic DNA using primers designed to span these splice sites yielded products whose sizes were consistent with the presence of introns, a conclusion that was confirmed by sequencing (data not shown). Genomic PCR was used to determine the structure of the *Tbx6r* genomic locus, which has an open reading frame encoded by 8 exons (Fig. 1B). The Genbank accession number for this sequence is EU926666. The presence of introns within the Tbx6r locus indicates that this gene arose through locus duplication, as in the case of the Tbx2/4 and Tbx3/5 clusters (Agulnik et al., 1996), rather than through genomic incorporation of a reverse transcription product, as in the case of MGA, which lacks T-box introns (Agulnik et al., 1996; Lardelli, 2003).

Since duplicated genes often retain the regulatory elements of the parent locus (McEwen et al., 2006), we compared the spatiotemporal expression of *Tbx6r* with *Tbx6* and *Tbx16*, the two Tbx6 sub-family members that are candidate ancestral loci of *Tbx6r* (Supplementary Fig. 1). The spatial and the temporal expression profiles of *Tbx6r* are both broadly similar to those of *Tbx6*, whereas there are distinct differences between *Tbx6r* and the combined expression patterns of the *Tbx16* products *VegT* and *antipodean*. These include the expression of both *Tbx6* and *Tbx6r* in the tailbud, in which domain *Tbx16* is absent. These comparative expression analyses suggest that *Tbx6r* arose through duplication of the *Tbx6* locus.

Despite the presence of 19 *X. laevis Tbx6r* ESTs in the NCBI database, no *X. tropicalis* orthologues were detected in this repository, so we investigated whether *Tbx6r* is present in the genomes of other members of the *Xenopodinae* by Southern analysis of genomic DNA derived from *X. laevis*, its congener *X. borealis,* and the more distantly-related *X. tropicalis* (Fig. 1C). To ensure that the probe was gene-specific, a region of the cDNA 3′ to the T-box was used as template, since BLAST searches of EST and genomic databases did not identify any other genes sharing homology with this region. As expected, a signal was detected in *X. laevis*. Bands were also detected in *X. borealis* and *X. tropicalis*, indicating that *Tbx6r* originated before the divergence of the *Xenopodinae* over 50 million years ago (Evans et al., 2004). Since Tbx6r is not present in the sequenced genomes of the zebrafish, pufferfish, mouse or human, the most parsimonious explanation is that it evolved at some point after amphibians branched off from the other vertebrate classes.

We investigated whether the *Tbx6r* locus is transcriptionally active in all three species by a comparative expression analysis. Genomic fragments encompassing the conserved T-box intron 3 (Wattler et al., 1998) from both *X. borealis* and *X. tropicalis* were cloned and sequenced to facilitate the design of intron-spanning primers for each species. RT-PCR of cDNA samples derived from both *X. borealis* and *X. tropicalis* produced amplicons corresponding to the size of a spliced product, which were distinguishable from the amplicons derived from genomic DNA (Fig. 1D). Sequencing of the *X. tropicalis* RT-PCR product confirmed that *Tbx6r* is expressed in this species and thus has not degenerated into a pseudogene (data not shown).

### Inductive properties of Tbx6r

Since Tbx6r probably evolved from Tbx6 locus duplication, we compared the biological activities of the two paralogues. An N-terminally truncated version of Tbx6r does not induce mesodermal markers but can induce anterior neural markers (Yabe et al., 2006). However, a truncated version of Xbra induces these markers artefactually (Rao, 1994), underscoring the importance of using the full-length protein in inductive assays. We therefore determined which of the first two methionines of the full-length Tbx6r is the preferred in vivo translational start site. Mutagenesis of M1 dramatically reduced translation of myc-tagged Tbx6r RNA in injected embryos, whereas mutation of M20 had no detectable effect (Supplementary Fig. 2A), indicating that translation in the embryo initiates predominantly from the first methionine that is absent from the previously published clone (Yabe et al., 2006). Full-length Tbx6r proved to behave like the truncated construct, inducing the anterior neural markers *Otx2*, *Pax6*, *Rx1* and *Zic1* (Supplementary Fig. 2B) without inducing either the mesodermal markers *Xbra* and *Vent1* or the endodermal marker *Sox17* in animal caps (Supplementary Fig. 2D). The inductive behaviours previously ascribed to the truncated version of Tbx6r (Yabe et al., 2006) are therefore representative of the divergent inductive properties of this protein, rather than being truncation artefacts. The inability of Tbx6r to induce mesoderm, and its capacity to induce anterior neural markers, together demonstrate the distinctive activity of this protein in comparison with its paralogue Tbx6. This functional divergence is striking, because both VegT and other more distantly-related T-box proteins, such as Xbra and Eomesodermin, can all induce mesoderm (Cunliffe and Smith, 1992; Lustig et al., 1996; Ryan et al., 1996; Uchiyama et al., 2001; Zhang and King, 1996), indicating that Tbx6r has relinquished an activity that has been conserved between several other members of the T-box family.

### Functional specificities of Tbx6r and Tbx6 reside within their C-termini

As Tbx6r and Tbx6 have distinct inductive properties, we created hybrid proteins to identify the regions of the proteins responsible for this functional specificity. The proteins were exchanged in the region immediately C-terminal to the T-domain (Fig. 2A). The hybrid protein Tbx6r-Tbx6 mimicked the inductive activity of Tbx6, inducing the mesodermal markers *Xbra* and *pmes* and also the endodermal marker *Sox17* in stage 11.5 animal caps, whereas the Tbx6-Tbx6r hybrid failed to induce these markers (Fig. 2B). This result demonstrates that the ability of Tbx6 to induce mesoderm resides in the region C-terminal of the T-domain, rather than in the DNA-binding domain. This is a surprising result, because the T-domain has been implicated in the functional specificity of the ascidian protein HrTbx6 (Takahashi et al., 2005). Conversely, the Tbx6-Tbx6r hybrid induced the anterior neural markers *Otx2*, *Rx1* and *Pax6*, in addition to the pan-neural markers *NCAM* and *Sox2* (Fig. 2C). This induction was very strong, exceeding the inductive capacity of wild-type Tbx6r, suggesting that the hybrid construct lacks some inhibitory regulatory region present in the native Tbx6r protein. In contrast, the Tbx6r–Tbx6 hybrid did not induce *Otx2* or *Rx1* but did activate *Pax6*, *NCAM* and *Sox2* at levels similar to those induced by wild-type Tbx6r, albeit less strongly than the Tbx6–Tbx6r construct (Fig. 2C). Since there is some level of neural induction by Tbx6r and both hybrid constructs, this capacity cannot be attributed solely to an activity residing within the Tbx6r C-terminus, whereas the equivalent region of Tbx6 is sufficient to bestow mesoderm-inducting activity.

Xbra3 has acquired a neural-inducing activity that is lacking from its close paralogue, Xbra (Strong et al., 2000). C-terminal truncations of Xbra convert this protein from a mesoderm inducer to an anterior neural inducer (Rao, 1994), reminiscent of the behaviour of Tbx6r. We investigated whether we could similarly change the inductive properties of Tbx6 by deleting the C-terminus of this protein, hypothesizing that the expression of a T-domain alone might exhibit antimorphic properties. Truncation of Tbx6 after the T-domain eliminated the capacity of this protein to induce mesoderm but did not result in the activation of anterior neural markers (Fig. 2D), indicating that ablation of the C-terminus is not sufficient to convert a T-box protein into a neural inducer, and allowing us to exclude the possibility that the C-terminus of Tbx6 is masking a latent neural-inducing activity residing in the anterior part of the protein.

### Evolution of regulatory control

Although the protein sequence of Tbx6r has diverged significantly from that of Tbx6, the expression patterns of the two genes are broadly similar. To investigate the extent to which regulatory controls have been conserved between *Tbx6* and *Tbx6r*, we examined the responsiveness of these genes to ligands expressed during early development. Both *Tbx6* and *Xbra* were induced in animal caps by a range of BMP4 concentrations, but expression of *Tbx6r* was not induced by this treatment (Fig. 3A). Similarly, *Tbx6r* did not respond to FGF ligands including bFGF, FGF4 and FGF8b (Fig. 3D), and was not activated by FGF8a, which induces neural markers in this assay (Fletcher et al., 2006). In contrast, both *Tbx6* and *Xbra* were up regulated by bFGF, FGF4 and FGF8b (Figs. 3B and C).

*Tbx6r* was up regulated by activin treatment. However, we note that this response was much less pronounced than the induction of *Xbra* and *Tbx6*, both in terms of the fold induction of the genes in response to ligand, and by the finding that *Tbx6r* is submaximally induced by 1 ng/ml activin (Fig. 3D). In contrast, *Xbra* and *Tbx6* are maximally induced at this concentration, since they show no further induction by 10 ng/ml activin (Figs. 3B and C).

These differences in ligand responsiveness between *Tbx6* and *Tbx6r* indicate that there have been significant evolutionary changes in the regulatory controls governing the two paralogues, with Tbx6r having lost much of the ligand responsiveness that has been conserved between evolutionary divergent members of the family such as *Xbra* and *Tbx6*. These results suggest that Tbx6r is a degenerate Tbx6 paralogue that has undergone significant regulatory drift. Our observations raise the question of how the two genes come to have such similar expression patterns, and a part of the explanation may come from the observation that Tbx6 can up regulate expression of Tbx6r (data not shown).

Further evidence for differences in the regulation of the two paralogues comes from a quantitative comparison of their expression in dorsal (DMZ) and ventral marginal zone (VMZ) explants. Expression of *Tbx6* and *Tbx6r* was measured in DMZ and VMZ explants that were isolated at early gastrula-stage 10.5 and harvested at late neurula stage 14. Whereas expression of *Tbx6* was enriched in ventral tissue, *Tbx6r* was elevated in dorsal explants (Fig. 4A). Therefore, despite the broadly similar expression patterns of *Tbx6* and *Tbx6r* observed in the developmental expression analysis of whole embryos (Supplementary Fig. 1) and referred to above, there are clear regional quantitative differences in the expression patterns of the paralogues in the presumptive mesoderm during early development.

FGF signalling is necessary for the activation of *Tbx6* (Fang et al., 2004), and we investigated whether *in vivo* expression of *Tbx6r* requires FGF signalling by treating marginal zone explants with the FGFR1 inhibitor SU5402. Expression of *Xbra* and *Tbx6* in both DMZ and VMZ explants was diminished in the absence of FGF signalling (Figs. 4B and C). *Tbx6r* expression in DMZ explants was greatly reduced in the presence of SU5402 (Fig. 4B) but there was no effect on expression of this gene in the VMZ (Fig. 4C). This result shows that although *Tbx6r* cannot be induced ectopically by FGF in the animal cap assay, its *in vivo* expression in the dorsal marginal zone is nevertheless ligand-dependent. The lack of effect of the inhibitor on *Tbx6r* expression in VMZs is further evidence of regulatory divergence between *Tbx6* and *Tbx6r*; Tbx6r expression in the ventral marginal zone may represent some residual, FGF-independent gene activity.

### Tbx6r function is required for *Xenopus* development

Over-expression experiments indicate that Tbx6r has functional activity, because it can induce neural markers in animal caps. However, such experiments do not indicate whether the gene is necessary for normal development. To address this question, we inhibited Tbx6r activity using antisense morpholinos, the target sites of which are marked on Fig. 5A. Three of these morpholinos (MO1, MO3, and MO4) gave phenotypes of varying severity, including axial abnormalities, anterior defects and reduced pigmentation, as shown in Figs. 5C–G. We were unable to rescue the morphant phenotype by over-expression of *Tbx6r* RNA; this is likely to be due to difficulties in titration, since over-expression of the RNA itself causes axial defects (data not shown). We experienced a similar problem with the attempted rescue of the Tbx6 morphants discussed at a later point in this paper, so we have controlled for morpholino specificity by the use of multiple morpholinos, including both translation-blockers and splice-blockers (Eisen and Smith, 2008).

Since MO1, MO2 and MO3 were designed prior to our characterisation of the complete genomic locus, we were initially confounded by the discovery that 30 ng MO3 could perturb development (Fig. 5F), even though its target sequence is distal to that of MO2, which is 3′ of the translational start site and has no phenotypic effects at this concentration (data not shown). Indeed, even injection of 90 ng MO2 caused only minimal effects (Fig. 5E), consistent with our assignation of ATG M1 as the translation start site. Genomic sequence analysis revealed that the more 3′ target site of MO3 falls on an exon–intron boundary and that a maximum of 12 bases of this morpholino can bind consecutively to the pre-mRNA. To ask whether MO3 perturbs *Tbx6r* splicing, we performed RT-PCR on cDNA derived from embryos injected with MO3, using intron-spanning primers (Fig. 5B). The size of the PCR product indicated that the first intron of Tbx6r was retained in injected embryos, and this was confirmed by sequencing the product, leading us to the surprising conclusion that splicing perturbations can be induced by a morpholino that targets solely exonic sequence.

The effectiveness of 50 ng MO4 as a *Tbx6r* splice inhibitor was confirmed by RT-PCR using intron-spanning primers (Fig. 5B). Only a small amount of the aberrant splice form was detected; it is likely that the bulk of this product underwent nonsense-mediated decay (Lewis et al., 2003). The target sequence of MO4 was quite conserved between *Tbx6r* and *Tbx6* (15/25 bases) but this morpholino did not affect Tbx6 splicing (Fig. 5B), thus demonstrating its specificity.

We confirmed that Tbx6r is functional in *X. tropicalis* as well as *X. laevis* by use of a splicing morpholino (Fig. 6). 30 ng XtTbx6r morpholino caused eye and pigmentation defects, and the shortened tails are consistent with the idea that there are abnormalities in posterior somite development (Figs. 6D and F). The phenotype is milder than that observed in *X. laevis* Tbx6r morphants (Figs. 5C–G) and in *X. tropicalis* Tbx6 morphants (Fig. 6E), perhaps because of incomplete knockdown by the XtTbx6r morpholino (Fig. 6A). There may also be differences in the relative expression and functionality of the two paralogues in each species, resulting from variations in quantitative partitioning of the ancestral locus function following divergence of the two lineages. However, the generation of phenotypes by Tbx6r depletion in both *X. laevis* and *X. tropicalis* demonstrates its necessity in both species, indicating that the fixation of this gene within the amphibian lineage occurred before the divergence of these species, at least 50 million years ago (Evans et al., 2004).

### Tbx6r is required for development of the paraxial mesoderm, intermediate mesoderm and neural crest

Embryos injected with morpholinos targeting Tbx6r completed gastrulation normally, expressing the mesodermal markers *Xbra* and *Wnt8* (Figs. 7A–F). No significant changes were evident in the expression of the myogenic marker *myoD* (Figs. 7G–I), but staining with the antibody 12/101, which recognizes skeletal muscle, indicated that somitic muscle differentiation was impaired (Figs. 7J and K), as was development of the intermediate mesoderm, revealed by loss of *Xlim1* expression (Figs. 7L–O).

Neural tissue was formed in embryos injected with Tbx6r morpholinos, although *sox3* was expressed in a relatively broad domain (Figs. 8A–C) and closure of the neural folds was delayed (data not shown). The anterior neural markers *otx2* and *pax6* were expressed at stage 16 (Figs. 8D–I), indicating that *Tbx6r* is not required for activation of these markers, even though it can induce them following over-expression in animal caps (Supp. Fig. 2B). *Tbx6r* is required for neural crest development, however, since expression of the neural crest markers *snail2* and *twist* was significantly reduced at neurula stages in embryos injected with morpholinos targeting Tbx6r (Figs. 8J–O; data not shown). Defects in neural crest migration and patterning persisted into tailbud stages, as shown by aberrant *snail2* expression (Figs. 8S and T) and loss of *twist* (Figs. 8U and V). Morpholino injection also inhibited expression of the hindbrain marker *krox20* during neurulation (Figs. 8P–R). In severe cases expression was lost in both rhombomeric stripes but in milder cases expression in rhombomere 3 (r3) persisted. In frogs, r5 contributes substantially to the neural crest, so loss of this marker is consistent with a neural crest phenotype. Morphologically, neural crest abnormalities were revealed by the loss of melanophores, which are derivatives of this cell type (Figs. 5C–G).

### Tbx6 is required for patterning the neural crest and intermediate mesoderm

Embryos lacking Tbx6r show defects in paraxial and intermediate mesoderm and neural crest. *Tbx6r* may have acquired these roles through neofunctionalization. Alternatively, they may represent ancestral functions of the locus that gave rise to Tbx6r. Since the intron positioning and expression pattern of *Tbx6* make it the best candidate for the parent locus of *Tbx6r*, we asked whether the two genes are required for similar developmental functions. The requirement for Tbx6 in patterning somitic musculature has been documented in both mice and *Xenopus* but this gene has not previously been implicated in the development of either the neural crest or the intermediate mesoderm. We noticed that *X. tropicalis* Tbx6 morphants have reduced pigmentation (Fig. 6E), suggesting that this gene is also necessary for neural crest development, so we performed a more detailed analysis in *X. laevis*.

To this end we cloned the first intron of the Tbx6 T-box and designed a splicing morpholino (T6sp1 MO) targeted at this exon–intron boundary. This morpholino, as well as one directed against the translational start site, caused shortened tails and pigmentation defects (Figs. 9B–E). 50 ng of T6sp1 severely depleted *Tbx6* mRNA levels without affecting *Tbx6r* splicing, as assessed by RT-PCR (Fig. 9A). In situ analysis of embryos with reduced levels of Tbx6 revealed neural crest defects similar to those observed in embryos with reduced Tbx6r, including a moderate reduction of *snail2* expression (Figs. 9 F–I) and loss of *krox20* in r5 at neurula stages (Figs. 9L and M), followed in tailbud stages by aberrant migration of *snail2*-expressing cells (Figs. 9J and K) and reduced *twist* expression (Figs. 9N and O). Intermediate mesoderm was also defective in embryos with reduced Tbx6, as assessed by the expression of *Xlim1* in tailbud stages (Figs. 9P–S). The diminution of neural crest and intermediate mesoderm in both Tbx6 and Tbx6r morphants suggests that these represent conserved functions of their last common ancestral locus rather than the acquisition of new functions by Tbx6r.

### Tbx6r induces FGF8 with distinct temporal dynamics from Tbx6-mediated ligand induction

Analysis of the phenotypes of embryos lacking Tbx6 and Tbx6r demonstrates that both genes are necessary for patterning of the neural crest. However, these genes are expressed in the mesoderm, rather than the ectoderm (Figs. 10A and B); (Uchiyama et al., 2001; Yabe et al., 2006) and are therefore likely to affect the neural crest in a non-autonomous manner, perhaps through the activation of a secreted protein, as is the case for the induction of wnt ligands by *Brachyury* orthologues in zebrafish (Martin and Kimelman, 2008).

We asked whether over-expression of either *Tbx6* or *Tbx6r* RNA could activate the genes encoding the mesodermally expressed ligands Wnt8, Wnt11, FGF4 and FGF8 in animal caps. Both Tbx6 and the hybrid construct Tbx6r–Tbx6 were able to induce all of these ligands in gastrula-stage animal caps (Fig. 10C), consistent with our earlier finding that the C-terminus of Tbx6 is important for its mesoderm-inducing activity (Fig. 2B). In contrast, neither Tbx6r nor the hybrid construct Tbx6–Tbx6r were able to induce these ligands in gastrula-stage caps, consistent with the previously demonstrated inability of Tbx6r to induce a variety of mesodermal markers (Yabe et al., 2006).

Surprisingly, Tbx6r was able to induce robust expression of FGF8 in animal caps cultured until stage 23 (Fig. 10D); the hybrid construct Tbx6–Tbx6r also induced FGF8 in these caps (data not shown). This effect was ligand-specific, since other ligands were not induced by Tbx6r (Fig. 10D). *FGF8* is likely to be a direct target of Tbx6, since a hormone-inducible Tbx6-VP16 construct can induce its expression within 1.5 h in the presence of cycloheximide in the animal cap assay (Li et al., 2006). Although Tbx6r lacks this capacity to up-regulate *FGF8* in a rapid manner, it has evidently retained some residual inductive ability, either through an indirect mechanism or through a weak auto-induction loop, because it can activate the gene after an extended culture period (Figs. 10C and D). We did not detect any major changes in *FGF8* expression by quantitative RT-PCR in embryos injected with Tbx6 or Tbx6r morpholinos (data not shown), even though this ligand is a known target of Tbx6–VP16 (Li et al., 2006). However, this analysis may not be sufficiently sensitive to detect localised down-regulation of gene expression, and is also complicated by the fact that we cannot discriminate between the relative expression of the *FGF8a* and *FGF8b* splice forms by this method.

### Differential induction of neural plate border markers by Tbx6r and Tbx6

The neural crest derives from the neural plate border. This region is located at the boundary between the neural plate and the non-neural ectoderm and gives rise to placodal ectoderm anteriorly and neural crest laterally (Hong and Saint-Jeannet, 2007). We asked whether Tbx6 and Tbx6r are capable of inducing neural plate border markers in animal caps. Both proteins induced the pan-placodal markers *six1* and *eya1*, demonstrating that these genes can bias uncommitted ectoderm towards a neural plate border fate. Tbx6 was a more potent inducer of these markers than Tbx6r and also activated *snail1* and *twist* (Fig. 10E), demonstrating that Tbx6 activity is sufficient for activation of neural crest specifier genes. Tbx6r did not induce these markers, indicating that the two proteins have differential abilities with regard to neural plate border specification. We tested the effects of the hybrid constructs on induction of neural plate border markers, finding that whereas both constructs induced placodal markers, only the Tbx6r–Tbx6 construct induced neural crest (Fig. 10E). Hence, the Tbx6r–Tbx6 construct mimics the behaviour of Tbx6 in terms of neural crest induction, demonstrating that this inductive property of Tbx6 resides within a region of the protein C-terminal to the T-box, as is the case for its capacity to induce mesoderm and endoderm (Fig. 10E).

### Tbx6 specifies neural crest cell fate through an FGF8- and Wnt8-dependent mechanism

As *FGF8* and *Wnt8* are Tbx6 targets (Fig. 10C) (Li et al., 2006), and have previously been implicated in neural crest formation (Monsoro-Burq et al., 2003; Monsoro-Burq et al., 2005), we investigated whether either of these secreted ligands is necessary for Tbx6-mediated neural crest induction. We co-expressed Tbx6-MT RNA in animal caps together with previously characterised morpholinos targeting FGF8 or Wnt8 (Fletcher et al., 2006; Li et al., 2006). Both morpholinos abrogated the induction of neural crest markers by Tbx6 (Fig. 10F). Together, our results show that in addition to its necessary role in neural crest induction (Fig. 9), Tbx6 over-expression is capable of specifying this cell fate in animal cap cells (Fig. 10E), and it does so through a ligand-dependent mechanism requiring both Wnt8 and FGF8 (Fig. 10F).

## Discussion

The T-box family has ancient origins, with members found throughout the Metazoa (Larroux et al., 2008; Yamada et al., 2007). Gene duplication has resulted in variations in T-box complement across the Metazoa but the locus of origin can usually be identified through sequence comparison, as is the case for the *Brachyury* duplicates that have arisen independently in *Xenopus*, zebrafish, amphioxus and *Hydra* (Bielen et al., 2007; Hayata et al., 1999; Holland et al., 1995; Martin and Kimelman, 2008; Strong et al., 2000), as well as the three Tbx6-type *dorsocross* genes in *Drosophila* (Reim et al., 2003). Since *Tbx6r* is not identified in the sequenced genomes of any other vertebrate class, it is likely to be the product of a relatively recent duplication event. Protein sequence comparisons and gene expression data suggest that *Tbx6r* arose as a result of *Tbx6* locus duplication, although there has been significant divergence of these loci, relative to the distances between other T-box duplicates.

### Structure-function analysis of Tbx6r

The divergent nature of Tbx6r is also reflected in its unusual inductive activity. Several T-domain proteins from divergent clades of the T-box family can induce mesoderm (Ryan et al., 1996; Smith et al., 1991; Stennard et al., 1999; Strong et al., 2000; Uchiyama et al., 2001), suggesting that this is an ancestral function of this group of proteins. It is therefore surprising that Tbx6r does not possess this activity and instead has acquired the ability to induce anterior neural tissue. This might occur through transcriptional repression, because a *Xenopus* Tbx6R-Engrailed repressor fusion construct has the same inductive capabilities as its parent protein (Yabe et al., 2006). However, Tbx6r is not the only T-domain protein capable of inducing neural tissue: Xbra3, a duplicate of Xbra in *Xenopus*, can induce posterior neural tissue while retaining its mesoderm-inducing activity (Strong et al., 2000), and the *Hydra* Brachyury homologue, Hybra2, resembles Tbx6r even more closely because it too induces anterior neural markers but not mesoderm in the animal cap assay (Bielen et al., 2007). A C-terminally truncated version of Xbra called B304 behaves similarly (Rao, 1994), suggesting that a latent neural-inducing activity within Xbra is normally masked by its C-terminus.

Experiments using chimeric proteins indicate that the C-terminus of Tbx6 is required for its inductive ability, because a construct lacking this domain cannot induce endoderm, mesoderm, or neural markers. This C-terminal region helps define the functional specificity of Tbx6 because when fused to the T-domain of Tbx6r it confers upon this chimeric protein the ability to induce mesoderm. This result contrasts with work in *Xenopus* and ascidians that has implicated the T-domain, rather than the C-terminus, in the definition of functional specificity (Conlon et al., 2001; Takahashi et al., 2005). It is, however, consistent with the observation that the different activities of the *Hydra* Brachyury orthologues reside within their C-termini (Bielen et al., 2007)*.* The ability of an N-terminal motif to restrict the inductive activity of Xbra is further evidence that regions outside the T-domain can modulate the biological activity of this protein (Marcellini et al., 2003; Messenger et al., 2005).

The C-terminus of Tbx6 is able to induce mesoderm when fused with a T-domain, but it is more difficult to assign the neural-inducing properties of Tbx6r to a particular region of the protein. The C-terminus of Tbx6r can activate neural genes when fused to the Tbx6 T-domain, but the T-domain of Tbx6r also exerts some influence, because a Tbx6r–Tbx6 construct can also induce some neural markers.

### Induction of *Tbx6* and *Tbx6r*

*Tbx6* and *Tbx6r* differ in the extent to which they are activated by ligands such as activin and FGF, with *Tbx6r* being much less responsive than *Tbx6* or the founder member of the T-box family, *Brachyury* (*Xbra*). These observations are noteworthy because *Xbra* and *Tbx6* diverged following an ancient duplication event, while the origin of *Tbx6r* represents a much more recent evolutionary event. The loss of ligand responsiveness by *Tbx6r* may have been facilitated by the retention of the ancestral locus, *Tbx6*, which can both respond to ligands and activate *Tbx6r*, thus providing an explanation for the similarities in their expression profiles, despite their differing ligand responsiveness. Tbx6r expression has not been completely freed of the influence of ligands, since its expression in the dorsal marginal zone is FGF-dependent. An ancient gene regulatory module that activates T-box genes through FGF signalling may operate within the dorsal mesoderm, since expression of *Xbra* and *Tbx6* in this region is also FGF-dependent.

### Loss-of-function analyses

Tbx6 is required for paraxial mesoderm formation in mice and amphibians (Chapman et al., 1996; Chapman et al., 2003; Tazumi et al., 2008) and our loss-of-function analysis demonstrates that Tbx6r is also involved in this process. However, our results also show that Tbx6 and Tbx6r are both required for development of the intermediate mesoderm. This tissue gives rise to the kidney, and expression of the pronephric marker *Xlim1* is abolished in both Tbx6r and Tbx6 morphants. In the chick embryo, pronephros formation in the intermediate mesoderm is influenced by signals derived from the paraxial mesoderm (Mauch et al., 2000), and our work suggests that these signals may be regulated by Tbx6 and Tbx6r; examination of intermediate mesoderm in mouse Tbx6 mutants will indicate whether this is an conserved function of Tbx6. FGF ligands may be among the signals regulated by Tbx6 and Tbx6r in the intermediate mesoderm because expression of *Xlim1* in the pronephros requires FGF signalling (Urban et al., 2006).

An unexpected finding of our work was that of the role of the Tbx6 sub-family in patterning the neural crest, the “fourth germ layer” (Hall, 2000). Both Tbx6 and Tbx6r morphants exhibited severe defects in neural crest formation, so this is probably an ancestral function that has been retained in both duplicates. Tbx6 and Tbx6r are both expressed in the paraxial mesoderm (Figs. 10A and B) (Uchiyama et al., 2001; Yabe et al., 2006), so they must pattern the neural crest via a non-autonomous mechanism, in the way that the Brachyury orthologues *no tail* and *bra* maintain Wnt ligand expression during zebrafish somitogenesis (Martin and Kimelman, 2008).

The paraxial mesoderm is necessary for neural crest induction (Bonstein et al., 1998; Monsoro-Burq et al., 2003), and it has been suggested that this interaction is mediated by FGF8 and Wnt8 (Monsoro-Burq et al., 2003; Monsoro-Burq et al., 2005). We note that Tbx6 can activate the expression of both *FGF8* and *Wnt8*, whereas Tbx6r can only induce *FGF8*, and these different ligand-inducing abilities may explain their different abilities to induce markers of the neural plate border. Thus, in the zebrafish embryo, FGF8 is necessary and sufficient for otic placode formation whereas Wnt8 is not required (Phillips et al., 2004). In contrast, both FGF8 and Wnt8 are required for neural crest induction (Hong et al., 2008). Our results are consistent with the suggestion that Tbx6r can activate placodal markers via FGF8, but cannot induce markers of neural crest specification through Wnt8. In contrast, Tbx6 can induce both FGF8 and Wnt8, allowing the activation of both placodal markers and neural crest specifiers. Consistent with this suggestion, we find that antisense morpholino oligonucleotides targeted against either FGF8 or Wnt8 prevent the induction of neural crest markers by Tbx6 in *Xenopus* animal pole regions.

## Conclusions

Our results identify new functions for Tbx6 in the development of the intermediate mesoderm and neural crest. In addition, we confirm and extend previous observations that Tbx6 plays a key role in paraxial mesoderm formation, activating secreted signals that cause overlying ectoderm to form neural crest. We also show that its paralogue, Tbx6r, is involved in the patterning of these tissues. This novel duplicate has retained functionality rather than degenerating into a pseudogene, augmenting the function of Tbx6 during development. The integration of Tbx6r into the Tbx6 gene regulatory network such that both genes are developmentally necessary represents an example of quantitative sub-functionalisation (Force et al., 1999; Postlethwait et al., 2004).

The involvement of Tbx6 sub-family members in neural crest formation may be an example of neofunctionalization within amphibians, because neural crest defects were not reported in the mouse *Tbx6* mutant (Chapman and Papaioannou, 1998). It is also possible that the Tbx6 sub-family has a more ancient and fundamental role in patterning the neural crest; a close analysis of this tissue type in Tbx6 sub-family mutants in other vertebrate classes will be of particular value in distinguishing between these hypotheses.

## Figures and Tables

**Figure:**
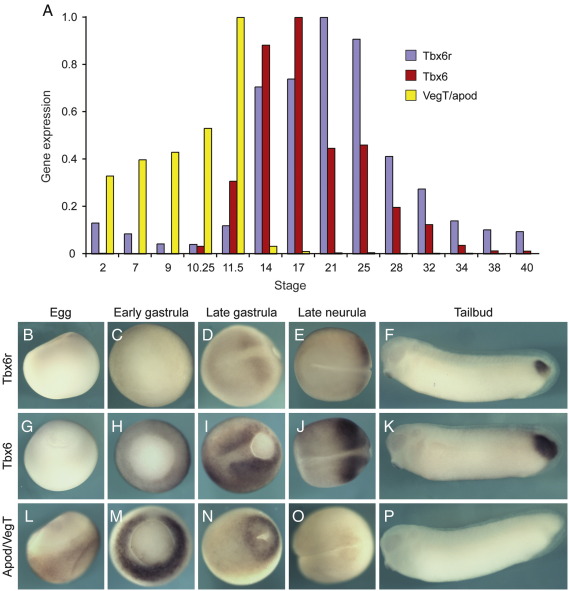


**Figure:**
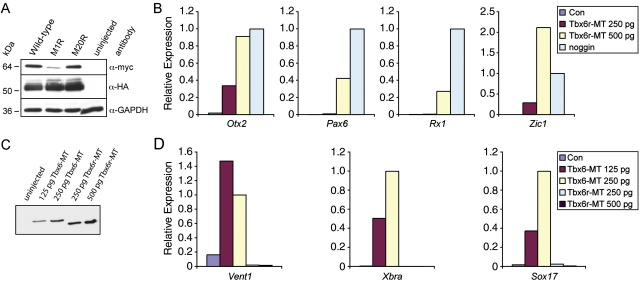


**Fig. 1:**
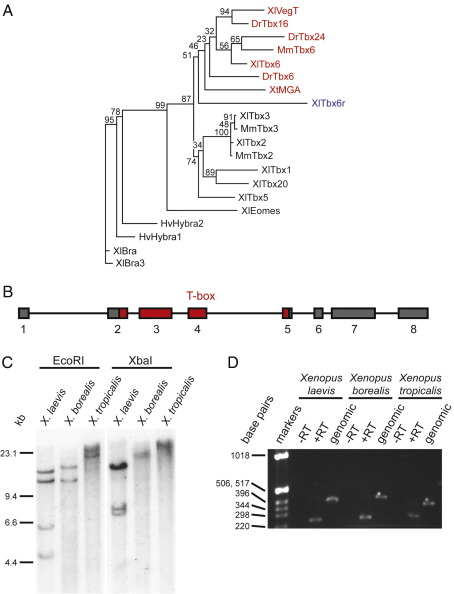
Genomic structure of Tbx6r, present and expressed in three frog species. (A) Unrooted phylogenetic tree created by PhyML based upon Tcoffee alignment of T-domains from *Xenopus laevis* (Xl), *X. tropicalis* (Xt), *Mus musculus* (Mm), *Danio rerio* (Dr) and *Hydra vulgaris* (Hv). Bootstrap values are shown. Red-labelled genes belong to the Tbx6 sub-family; Tbx6r is labelled in blue. (B) Genomic locus encompassing Tbx6r open reading frame. (C) Genomic Southern blot probed with Tbx6r detects bands in three different species of Xenopodinae. (D) RT-PCR detects expression of Tbx6r mRNA in all three species. The cDNA amplicons from all three species (+ RT lanes) are of the expected size of 248 base pairs (bp). Genomic DNA was included as a positive PCR control.

**Fig. 2:**
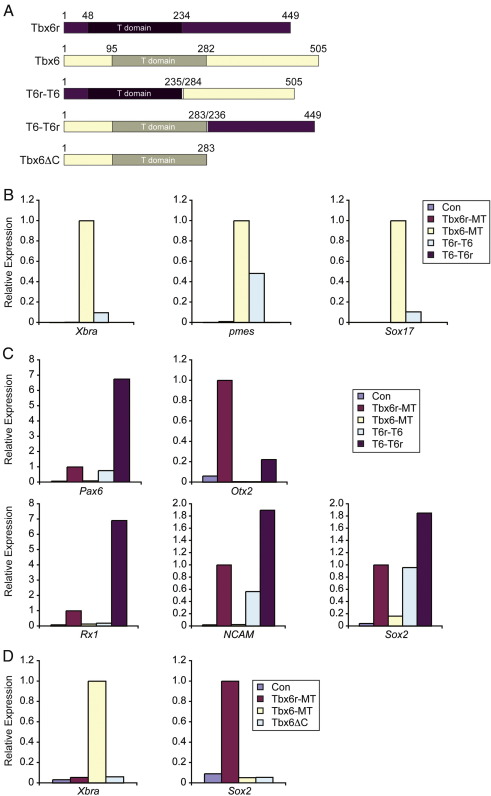
Hybrid proteins implicate the C-termini of Tbx6 and Tbx6r in functional specificity. (A) Diagram of the hybrid constructs relative to the parent proteins. A 3 amino acid linker joins the two fragments. (B) Mesoderm induction in mid gastrula-stage 11.5 animal pole regions. (C) Neural induction in stage 23 animal caps. 500 pg each of Tbx6r-MT, Tbx6–MT, Tbx6r–Tbx6 and Tbx6–Tbx6r RNAs were injected, and uninjected control caps were collected for comparison. All experiments were conducted three times except D, which was performed twice; representative results of individual experiments are shown.

**Fig. 3:**
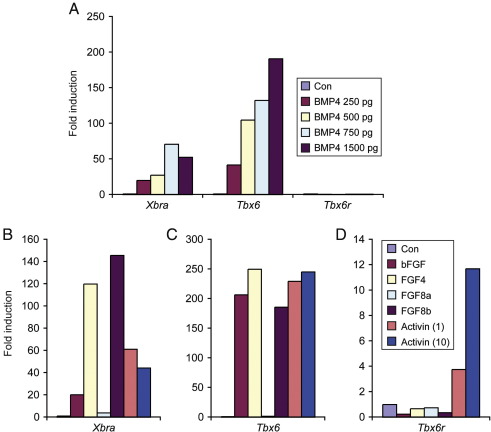
Comparative responsiveness of T-box genes to ligands. (A) Effect of increasing amounts of injected BMP4 mRNA on the expression of Xbra, Tbx6 and Tbx6r, compared with uninjected control animal caps. (B) Induction of Xbra, Tbx6 (C) or Tbx6r (D) in response to 100 ng/ml of various FGFs, and to 1 ng/ml and 10 ng/ml activin. All caps were collected at stage 18. A single experiment is depicted; results were duplicated in independent experiments. Control caps were not treated with ligands.

**Fig. 4:**
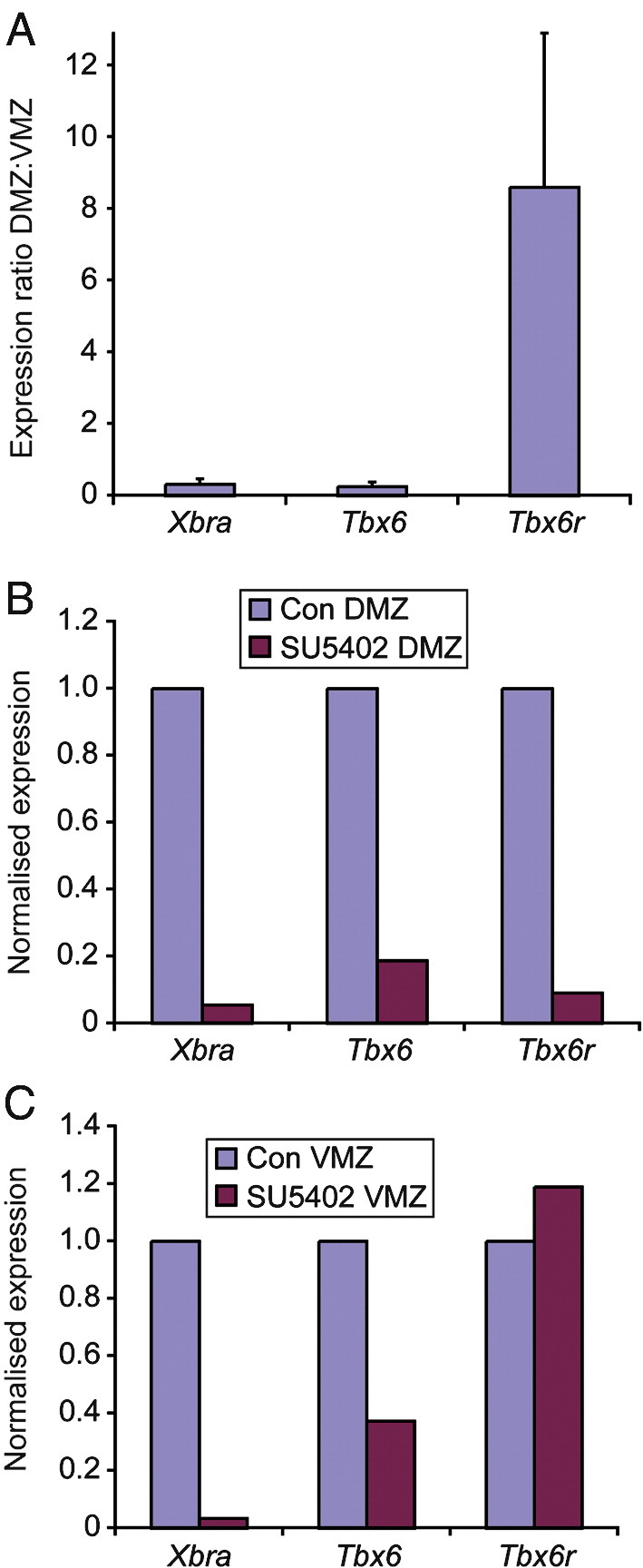
Differential requirements for FGF signalling in the maintenance of T-box expression. (A) Comparison of T-box expression levels in dorsal versus ventral marginal zone explants collected at stage 14. Graphs show the mean and standard error values of four experiments. (B) Relative expression of T-box genes in untreated and SU5402-treated dorsal marginal zones (DMZ) and (C) ventral marginal zones (VMZ). Results of the representative experiments shown in B and C were replicated in triplicate.

**Fig. 5:**
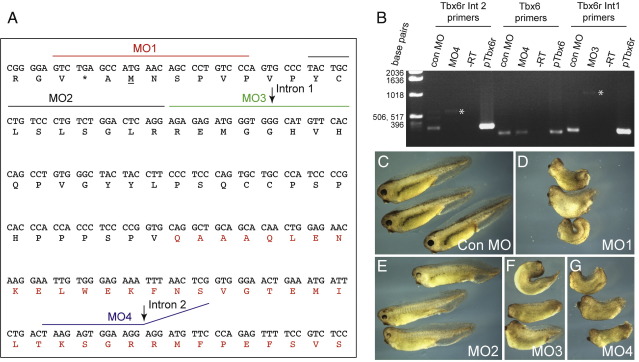
Tbx6r depletion using antisense morpholino oligonucleotides causes developmental defects in *X. laevis*. (A) Diagram illustrating positions of the various morpholinos used relative to the Tbx6r mRNA. The translational initiation site is underlined and the T-domain is highlighted in red. MO3 is an exact match to the RNA sequence because it was designed as a translation blocking morpholino although it acts as a splice-blocker, whereas MO4 is an inexact match because the 3′ region targets intronic sequence. (B) RT-PCR analysis of splicing morpholino efficacy and specificity in stage 19 embryos injected with 50 ng morpholino. Gene-specific primers span the indicated introns (Int). Asterisks indicate unspliced products. pTbx6 and pTbx6R are positive control PCR products generated from plasmid DNA. (C–E) Embryos injected with 90 ng of indicated morpholino. (F, G) Embryos injected with 30 ng morpholino.

**Fig. 6:**
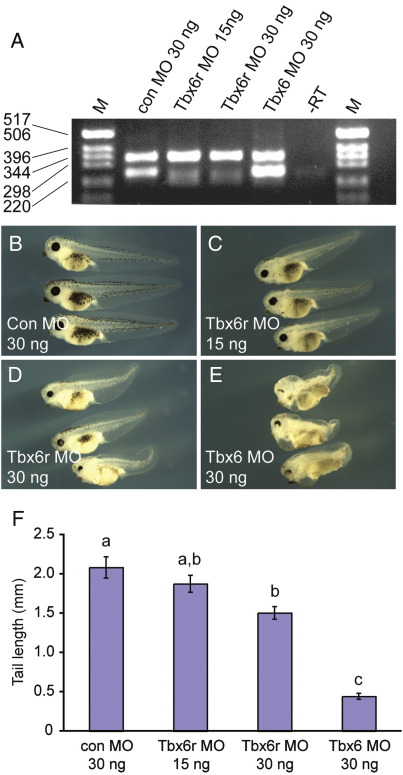
Phenotypic defects resulting from depletion of Tbx6r and Tbx6 in *X. tropicalis.* (A) Efficacy of splicing morpholino in siblings of embryos pictured in B–E. The upper band is of a size consistent with an unspliced product and may represent pre-mRNA since it is present in all samples. XtTbx6r MO inhibits splicing significantly but incompletely. Tbx6r splicing is not affected in Tbx6 morphants. Embryos were injected at the one-cell stage with the following morpholinos: 30 ng GeneTools control (B), 15 ng XtTbx6r (C), 30 ng XtTbx6r (D), 30 ng XtTbx6 (E). Tail lengths were measured from the proctodeal opening to the tail tip at stage 41. Mean values ± the standard error of the mean are shown in (F). Measurements were subjected to ANOVA followed by Scheffe's Test of Least Significant Difference. The annotations a, b and c above the bars on the graphs represent statistically significant differences between groups at *p* < 0.05.

**Fig. 7:**
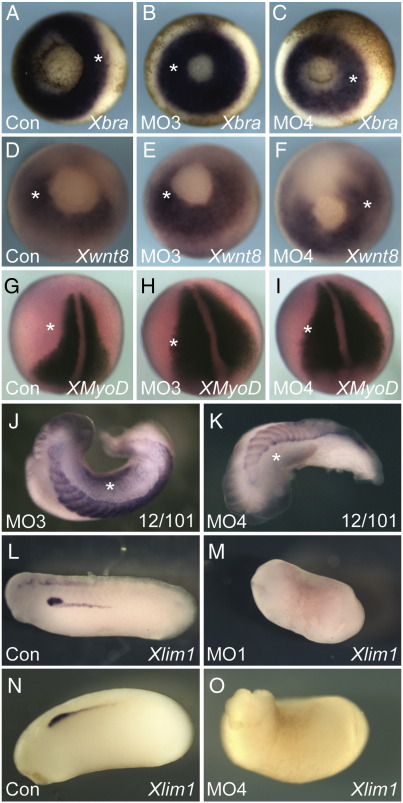
Mesoderm patterning in Tbx6r-depleted embryos. Embryos were injected unilaterally at the 2-cell stage with 25 ng of the indicated morpholino. The probes used are indicated on the images. All images are of in situ hybridisations except J and K, which are antibody stains. Asterisks indicate injected sides. (A–F) vegetal views of gastrulae; (G–I) dorsal views of neurula; (J–K) dorsal views of tailbud embryos, anterior to left. (L–O) are lateral views of hemi-injected tailbud embryos.

**Fig. 8:**
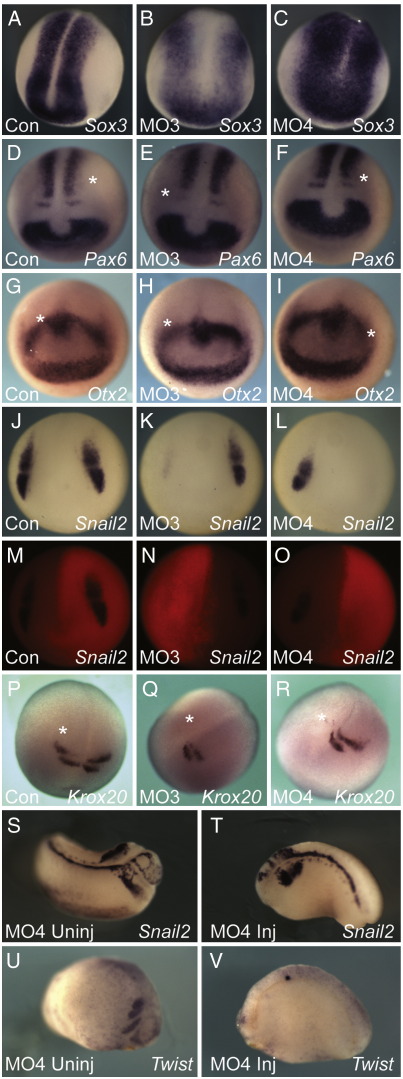
Neural patterning in Tbx6r-depleted embryos. All embryos were unilaterally injected at the 2-cell stage with 25 ng indicated morpholino except A–C, which were injected with 50 ng at the 1-cell stage. The morpholinos were lissamine-labelled to allow determination of injected side marked by an asterisk (M–O are fluorescent images of the embryos in J–L). In situ probes used are indicated on the figures. (A–C) dorsal views of neurula, anterior at bottom; (D–R) anterior views of neurula; (S–V) lateral views of tailbud embryos; T, V are injected sides of embryos in S, U respectively.

**Fig. 9:**
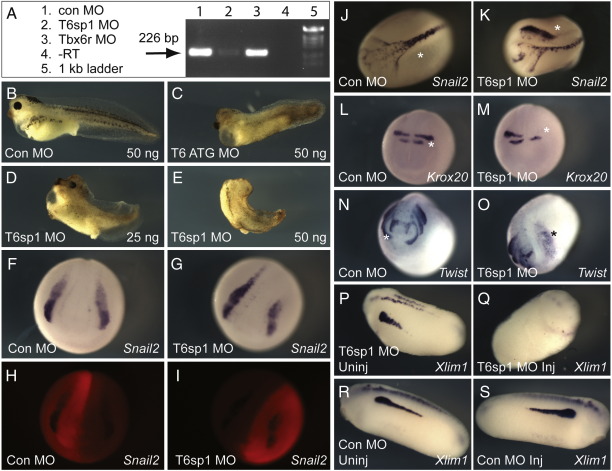
Embryos lacking Tbx6 have defects in neural crest and paraxial mesoderm formation. (A) Test of Tbx6 splicing morpholino (T6sp1) efficacy and specificity by RT-PCR. The 226 bp amplicon indicating correct splicing of Tbx6 is greatly reduced in stage 16 embryos injected with 50 ng T6sp1 MO but unaffected in siblings injected with 50 ng Tbx6r MO4. (B–E) Morphant phenotypes of Tbx6 translation-blocker (T6 ATG MO) and splice-blocker (T6sp1 MO) morpholinos. (F–R) In situ analysis of embryos unilaterally injected (*) with 25 ng T6sp1 MO at the 2-cell stage. F, J, L, N were injected with control morpholino; G, K, M, O were injected with T6sp1 MO. H and I are fluorescent images of the anterior views of the neurula depicted in F and G respectively, showing distribution of the lissamine-labelled morpholino. P and Q show uninjected and T6sp1 MO-injected sides of the same tailbud embryo; R and S are the equivalent images of a control-injected morphant. J, K: dorsal views; L–O anterior views.

**Fig. 10:**
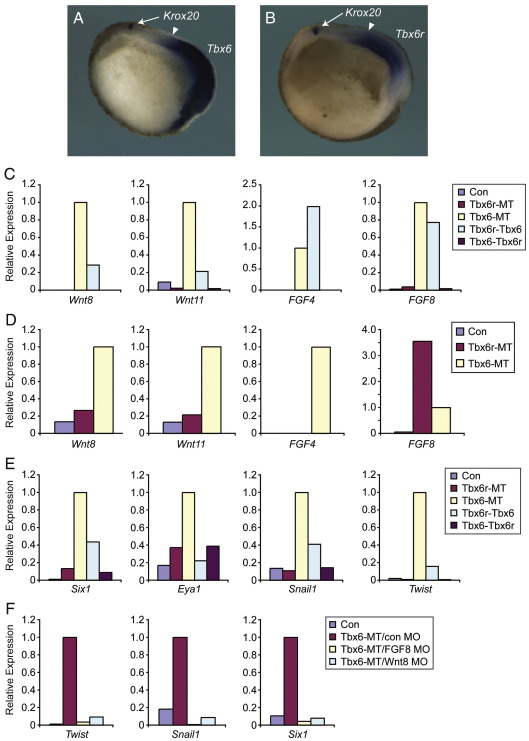
Tbx6 and Tbx6r induce FGF8 and neural plate border markers. (A) Tbx6 expression and (B) Tbx6r expression in the mesoderm of parasagittally bisected stage 15 embryos co-stained with the neurectodermal marker Krox20. (C) Differential ligand-inducing activities of Tbx6, Tbx6r and the hybrid constructs in gastrula-stage caps. 500 pg of each RNA was injected. (D) FGF8 is induced by 500 pg Tbx6r–MT in stage 23 caps. (E) Neural plate border induction in stage 23 animal caps. (F) Inhibition of Tbx6-mediated neural plate border induction by 500 pg Tbx6–MT RNA in stage 23 animal caps by 50 ng morpholino targeting either FGF8 or Wnt8. Experiments were independently duplicated (C, D, F) or triplicated (E); representative results of individual experiments are shown. Control caps in (C) to (F) were derived from uninjected embryos.
