# Supplementary material for: A divergent Tbx6-related gene and Tbx6 are both required for neural crest and intermediate mesoderm development in Xenopus
Source: Dev Biol. 2010 Apr 1;340(1):75–87. doi: 10.1016/j.ydbio.2010.01.013 (PMC2877776; doi:10.1016/j.ydbio.2010.01.013)
Supplement: Supplementary file 1 [file mmc1.doc]

**Supplementary Fig. 1.** Comparative spatiotemporal expression analysis of *Tbx6r*, *Tbx6* and *VegT/apod*. (A) Temporal analysis of the expression of the three genes during *X. laevis* development measured by quantitative RT-PCR. (B-P) Spatial analysis of gene expression by in situ hybridisation. B, G, L: side view; C, H, M: vegetal view; D, E, I, J, N, O: dorsal view, anterior to left; F, K, P: lateral view, anterior to left.

**Supplementary Fig. 2.** The biological activity of Tbx6r is atypical of the Tbx6 sub-family. (A) Determination of translational start codon by western analysis. Lanes contain lysates from early gastrula stage 10 embryos injected at the 1-cell stage with 500 pg RNA made from the wild-type myc-tagged Tbx6r-MT construct or from derivatives in which either of the first two methionines were mutated (M1R and M20R). All injected samples were spiked with 500 pg Xbra-HA as translation control and the blot was probed sequentially with anti-myc, anti-HA and anti-GAPDH antibodies, the last of these acting as a loading control. (B) Expression of anterior neural markers in animal caps cultured to stage 23 with the RNA amount injected into the 1-cell embryo specified in picograms. Expression in all samples was normalised to that of *ornithine decarboxylase* (*ODC*) as was the case for all subsequent experiments. The animal cap assay was performed three times and results of a representative experiment are shown. Here, the level of induction depicted is relative to that caused by 400 pg noggin RNA. (C) Comparison of translation efficiency of the Tbx6r-MT and Tbx6-MT constructs in NF10 embryos; siblings to those shown in D. (D) Expression of mesodermal and endodermal markers in NF 11.5 animal caps. Induction is depicted relative to that caused by 250 pg Tbx6. Control caps in (B) and (D) were derived from uninjected embryos.
